# Disrupted Neural Activity in Individuals With Iridocyclitis Using Regional Homogeneity: A Resting-State Functional Magnetic Resonance Imaging Study

**DOI:** 10.3389/fneur.2021.609929

**Published:** 2021-02-12

**Authors:** Yan Tong, Xin Huang, Chen-Xing Qi, Yin Shen

**Affiliations:** ^1^Eye Center, Renmin Hospital of Wuhan University, Wuhan, China; ^2^Department of Ophthalmology, Jiangxi Provincial People's Hospital, Nanchang, China; ^3^Frontier Science Center for Immunology and Metabolism, Medical Research Institute, Wuhan University, Wuhan, China

**Keywords:** iridocyclitis, regional homogeneity, resting-state fMRI, spontaneous brain activity, inflammation

## Abstract

**Objective:** This study used the regional homogeneity (ReHo) technique to explore whether spontaneous brain activity is altered in patients with iridocyclitis.

**Methods:** Twenty-six patients with iridocyclitis (14 men and 12 women) and 26 healthy volunteers (15 men and 11 women) matched for sex and age were enrolled in this study. The ReHo technique was used to comprehensively assess changes in whole-brain synchronous neuronal activity. The diagnostic ability of the ReHo method was evaluated by means of receive operating characteristic (ROC) curve analysis. Moreover, associations of average ReHo values in different brain areas and clinical characteristics were analyzed using correlation analysis.

**Result:** Compared with healthy volunteers, reduced ReHo values were observed in patients with iridocyclitis in the following brain regions: the right inferior occipital gyrus, bilateral calcarine, right middle temporal gyrus, right postcentral gyrus, left superior occipital gyrus, and left precuneus. In contrast, ReHo values were significantly enhanced in the right cerebellum, left putamen, left supplementary motor area, and left inferior frontal gyrus in patients with iridocyclitis, compared with healthy volunteers (false discovery rate correction, *P* < 0.05).

**Conclusion:** Patients with iridocyclitis exhibited disturbed synchronous neural activities in specific brain areas, including the visual, motor, and somatosensory regions, as well as the default mode network. These findings offer a novel image-guided research strategy that might aid in exploration of neuropathological or compensatory mechanisms in patients with iridocyclitis.

## Introduction

Uveitis is the most common type of inflammatory ophthalmological disease and has been estimated to cause up to 10% of legal blindness in the USA (1). Iridocyclitis is an acute inflammation of the iris and ciliary body; this is the most common pattern of uveitis, which is present in 85% of affected patients. Typical clinical features of iridocyclitis include eye redness, pain, blurred vision, photophobia, and miosis (2). HLA-B27 is a common risk factor for anterior uveitis, which has been found in ~40–70% of patients with uveitis (3). A range of complications such as secondary glaucoma, high intraocular pressure, cystoid macular edema, and posterior synechiae often occur, especially in patients with HLA-B27 (4). Subsequently, visual acuity can decrease temporarily or permanently because of the underlying inflammatory process or ocular complications of iridocyclitis. Moreover, a variety of patients with non-infectious iridocyclitis exhibit immune-mediated diseases (5), such as ankylosing spondylitis (AS), interstitial nephritis, and sarcoidosis. Elucidation of the underlying etiology may be challenging, because there is considerable variability in these mechanisms (e.g., from infectious to autoimmune diseases); however, this elucidation remains important, especially for patients with recurrent iridocyclitis.

Over the past few years, extensive neuroimaging researches have been conducted to evaluate cortical structural abnormalities in patients with iridocyclitis. Multiple studies have recorded ocular morphologic alterations affected by uveitis, including alterations in peripapillary retinal nerve fiber layer thickness, macular volume, and retinal thickness (6–8). Both cellular and humoral responses to a series of retinal antigens and their epitopes are known to occur in patients with iridocyclitis (9). Thus, even mild ocular inflammation can affect the ocular posterior segment, potentially leading to retinal and brain neurodegeneration through the visual pathway (10). Moreover, the retinal vessels have the same physiological, anatomical, and embryological characteristics with cerebral vessels; various quantitative and qualitative alterations involving the retinal capillary plexuses or choriocapillaris have been observed in patients with uveitis by means of optical coherence tomography angiography (11, 12). In addition, patients with iridocyclitis were shown to have a greater risk of depression and tend to adopt negative coping strategies (13, 14). In a pilot clinical study, Maca et al. pointed out that patients with iridocyclitis showed symptoms of cognition impairment including cognitive avoidance, distraction, and self-revalorization deficit through standardized psychological questionnaires (15). However, the abovementioned studies were limited to analyses of neuronal morphological changes and structural abnormalities in patients with iridocyclitis. To our knowledge, there is a lack of direct evidence regarding altered brain function in patients with iridocyclitis. Here, we hypothesized that iridocyclitis would influence the functions of certain brain areas, which might facilitate identification of the underlying neural mechanism.

Resting-state functional magnetic resonance imaging (fMRI) permits visualization of functional changes in the whole brain *in vivo*; it has the advantage of non-invasiveness, accurate positioning, and no ionizing radiation (16). Synchronous neuronal activity has been shown to occur in the normal human brain, particularly during memory and learning, in previous fMRI and electroencephalographic studies (17, 18). Moreover, transmission of synchronous neuronal activity is known to be involved in neuronal information processing (19). Regional homogeneity (ReHo) is a highly reliable fMRI index for evaluation of local synchronous neural activity patterns at rest; it measures the coherence of the blood oxygen level-dependent signal between the time series of a given cluster and its nearest neighbors by using Kendall's coefficient of concordance (20). The principle of the blood oxygen level-dependent signal is based on the inconsistencies in the local hemodynamics of neurons following excitation, in order to reveal spontaneous neuronal activity by quantifying alterations in blood oxygen level signals (21). Areas with higher ReHo signals imply that those brain regions have similar activities, compared with their neighbors. In contrast to conventional seed-based functional connectivity technique, ReHo provides the possibility to search for abnormalities in the entire brain functional connectome without pre-definition the region of interests. Besides, it is more stable than amplitude of low-frequency fluctuation method and less affected by global nuisances in the retest analysis (22). Most previous researches have proven that the ReHo technique is a reliable technique for application in various neuro-ophthalmological assessments; it has been widely used to reveal the mechanisms of ophthalmologic diseases including diabetic retinopathy (23), optic neuritis (24), amblyopia (25), and retinal detachment (26).

Iridocyclitis can induce both structural and functional alterations to the retina and its vessels, thereby affecting visual function. fMRI can be used to visualize alterations in regional neuronal activity and serve as a valuable monitoring modality, thereby improving disease management. Thus far, the spontaneous brain activity patterns of patients with iridocyclitis has been unclear. This study applied the ReHo technique to investigate spontaneous neuronal activity in patients with iridocyclitis.

## Methods

### Participants

The study followed the tenets of the Declaration of Helsinki and was approved by the institutional review board of the Eye Center, Renmin Hospital of Wuhan University. Each participant provided signed informed consent to participate in our study. This study enrolled 26 patients with iridocyclitis (mean age 45.15 ± 14.95) and 26 healthy volunteers (mean age 45.30 ± 13.87) who were matched on the basis of sex, age, and education. Patients with iridocyclitis enrolled in the study met the inclusion criteria as follows: [1] diagnosis of iridocyclitis, based on the Standardization of Uveitis Nomenclature Working Group classification (27); [2] ability to undergo magnetic resonance imaging scanning; [3] right-handed preference; and [4] no history of psychotropic drug use or psychiatric diseases. The exclusion criteria for patients with iridocyclitis were as follows: [1] presence of other ocular diseases (e.g., age-related macular degeneration, high myopia, epiretinal membrane, and glaucoma); [2] history of refractive/vitreoretinal surgery or ocular trauma; and/or [3] systemic diseases.

Inclusion criteria for healthy volunteers were as follows: [1] no retinal diseases such as diabetic retinopathy, cataract, or macular edema; [2] no psychiatric or neurological disorders; [3] no contraindications for magnetic resonance imaging scanning; [4] right-handed preference; and [5] binocular visual acuity ≥ 1.0. All participants underwent a complete ophthalmic assessment (biomicroscopy, slit-lamp examination, best-corrected visual acuity measurement, fundus examination, indirect ophthalmoscopy, and fluorescein angiography).

### MRI Parameters

Both whole-brain functional and T1-weighted MRI scans were carried out on a 3.0T GE MR750W scanner (GE Healthcare) with a standard head coil. Each participant was instructed to stay awake with eyes closed and relax their minds until the examination was over (28, 29). The whole-brain anatomical T1-weighted images were collected with a three-dimensional spoiled gradient-recalled echo sequence with following parameters: repetition time (TR)/echo time (TE), 8.5 ms/3.3 ms; gap, 0 mm; field of view (FOV), 240 × 240 mm^2^; acquisition matrix, 256 × 256; thickness, 1.0 mm; and flip angle, 12°.

The whole-brain fMRI data was recorded by applying gradient-recalled echo-planar imaging sequence with parameters as follows: TR/TE, 2,000 ms/25 ms; gap, 1.2 mm, thickness, 3.0 mm; FOV, 240 × 240 mm^2^; acquisition matrix, 64 × 64; 35 axial slices; and flip angle, 90°. The whole scanning time was ~15 min, and a total of 240 volumes of functional images were acquired.

### fMRI Data Preprocessing

Initially, functional images were checked by the MRIcro software (http://www.MRIcro.com) to exclude unqualified data. All preprocessing was performed using the Data Processing & Analysis of Brain Imaging (DPARSFA4.3, Institute of Psychology, Beijing) and the Statistical Parametric Mapping 12 (The MathWorks, Inc.) software running on Matlab 2014b (MathWorks, Natick, MA, USA) (30). [1] Original DICOM files were converted into NIFTI files. [2] The first 10 volumes of each functional time series were discarded to maintain magnetization equilibrium. [3] The remaining 230 volumes of functional images were modified for slice timing effects, motion corrected, and realigned. Data from subjects whose head motion with maximum displacement in any axis of >2.0 mm or head rotation of >1.5° were excluded. [3] Individual T1-weighted images were registered to the mean fMRI data, and then, the resulting aligned T1-weighted images were segmented using the Diffeomorphic Anatomical Registration Through Exponentiated Lie Algebra toolbox for improving spatial precision in the normalization of fMRI data (31). All the data were ultimately normalized to the standard Montreal Neurological Institute (MNI) space. [4] Detrend of the time course was performed. [5] Linear regression analysis was used to remove nuisance covariates (such as white matter signal, six head motion parameters, and cerebrospinal fluid signal); [5] After that, the fMRI images were band pass-filtered (0.01–0.08 Hz) to reduce the effects of low-frequency drift and high-frequency signals (32).

### ReHo Calculation

Calculation of ReHo values for fMRI data was conducted using REST (http://www.restfmri.net) toolbox. ReHo reflects the local synchronization between the spontaneous activity of a given voxel and its nearest neighboring voxels (20). ReHo is calculated by Kendall's coefficient of concordance with the formula below, where *W* represents the Kendall's coefficient of concordance among given voxels; *R*_*i*_ represents the sum rank of the time point; *n* represents the number of ranks; *K* = 27; and *R* = (*n* + 1) *K* / 2 represents the mean of the *R*_*i*_s. To reduce the impact of individual variability, the ReHo index was divided by the global the mean ReHo value. Finally, the fMRI data were smoothed with a 4 × 4 × 4 mm^3^ full-width at half-maximum Gaussian kernel.

$$\begin{array}{l} W=\frac{\sum {\left(R_{i}\right)}^{2}-n{\left(\bar{R}\right)}^{2}}{\frac{1}{12}K^{2\left(n^{3}-n\right)}} \end{array}$$

### Statistical Analysis

Differences between participants' demographic and clinical variables were analyzed by independent sample *t*-tests or the chi-square test using SPSS software (SPSS version 20.0, IBM Corporation, Armonk, NY, USA). *P* < 0.05 was considered to imply statistical significance. Besides, values are displayed as the mean ± standard deviation. Multiple comparison correction was conducted using the false discovery rate (FDR) method, and the statistical threshold for significance was set at *P* < 0.05. All ReHo maps were *z*-transformed with Fisher' *r*-to-*z* transformation to reduce the impacts of individual variations for group statistical comparisons. One-sample *t*-tests were performed to evaluate patterns of *z*-value ReHo maps, and two-sample *t*-tests were performed to investigate differences in ReHo values between patients with iridocyclitis and the healthy volunteers using SPM 8 software. The specific anatomical locations of all statistically significant results were presented as an image by BrainNet Viewer software (https://www.nitrc.org/projects/bnv/).

Pearson's correlation coefficients were calculated to investigate possible relationships between the average *z* ReHo of different brain areas and clinical characteristics of patients with iridocyclitis using SPSS software. In addition, brain areas with a distinctly different mean ReHo values between patients with iridocyclitis and healthy volunteers were analyzed using the receiver operating characteristic (ROC) curve method (SPSS version 20.0).

## Results

### Demographics and Clinical Data

**Figure 1:**
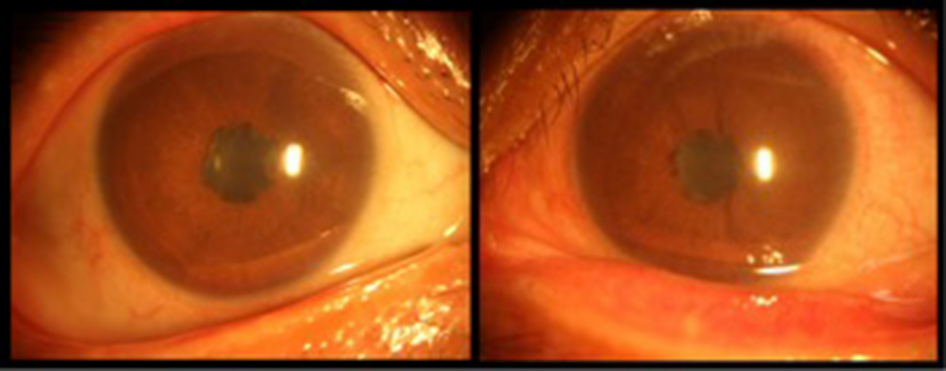
Typical anterior segment photograph of iridocyclitis.

The typical anterior segment photograph of iridocyclitis is displayed in Figure 1. There were no significant differences in age (*P* = 0.97), sex (*P* = 0.780), and educational status (*P* = 0.871) between the iridocyclitis group and healthy volunteer group. By contrast, the notable differences were observed in the best-corrected visual acuity-left and best-corrected visual acuity-right (*P* < 0.001) between the two groups. More participants' demographic data are presented in Table 1.

**Table 1:** General clinical information of patients with iridocyclitis and healthy volunteers.

	**Iridocyclitis group**	**HC group**	***T*-values**	***P*-values**
Sex (male/ female)	14/12	15/11	N/A	0.780
Mean age (years)	45.15 ± 14.95	45.30 ± 13.87	−0.038	0.970
Education (years)	10.96 ± 3.73	11.12 ± 2.86	−0.164	0.871
BCVA-OD	0.44 ± 0.27	1.16 ± 0.16	−11.474	<0.001^*^
BCVA-OS	0.43 ± 0.37	1.19 ± 0.16	−9.352	<0.001^*^
Handedness	26 R	26 R	N/A	N/A
Diagnosis of iridocyclitis (right eye/left eye)	11/15	N/A	N/A	N/A

*Chi-square test for sex. Independent t-test was used for other normally distributed continuous data. Data are presented as mean ± standard deviation. Abbreviations: HC, healthy control; BCVA, best-corrected visual acuity; OD, oculus dexter; OS, oculus sinister; N/A, not applicable; R, right*.

* *indicates statistically significant*.

### ReHo Differences

**Figure 2:**
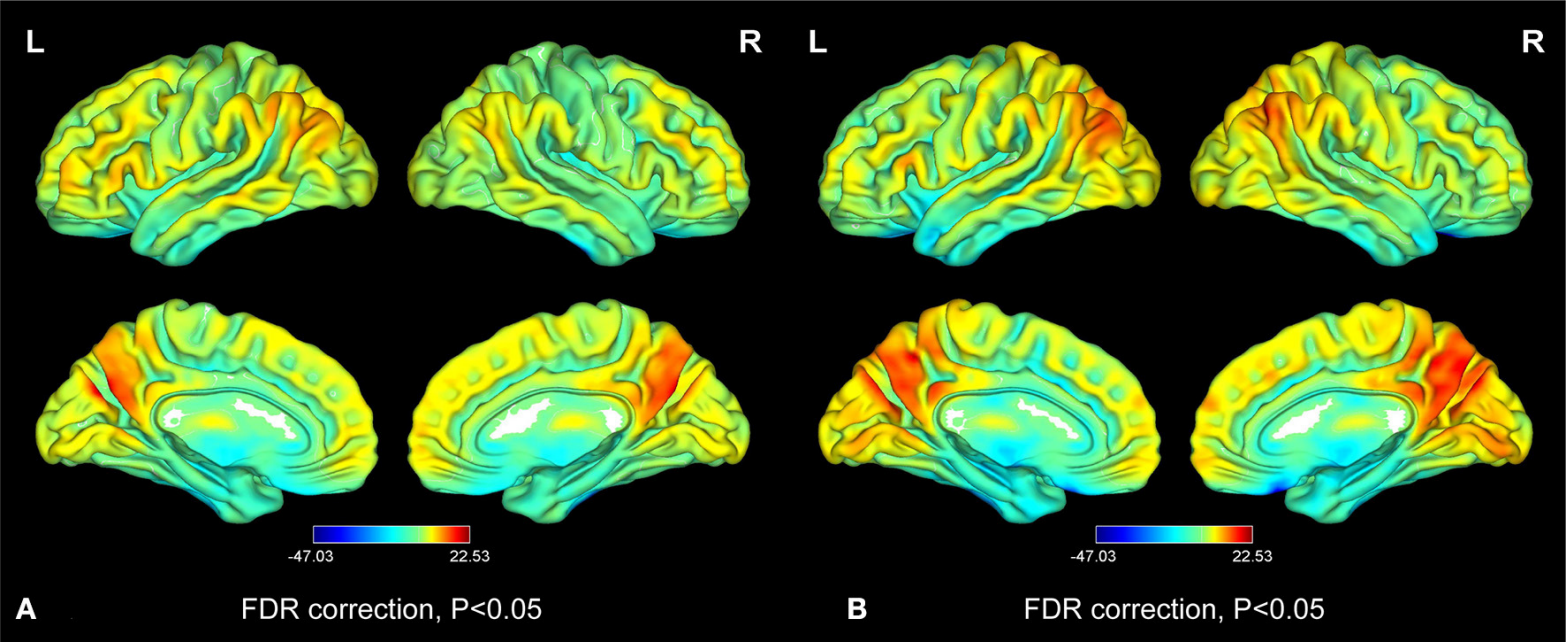
Distribution patterns of the ReHo value at the group level in iridocyclitis patients and healthy volunteers. One-sample *t*-test result of ReHo maps within the iridocyclitis **(A)** and healthy volunteers **(B)**. The color bar represents the *t*-values (FDR correction, *P* < 0.05). Abbreviations: L, left; R, right; FDR, false discovery rate correction.

**Figure 3:**
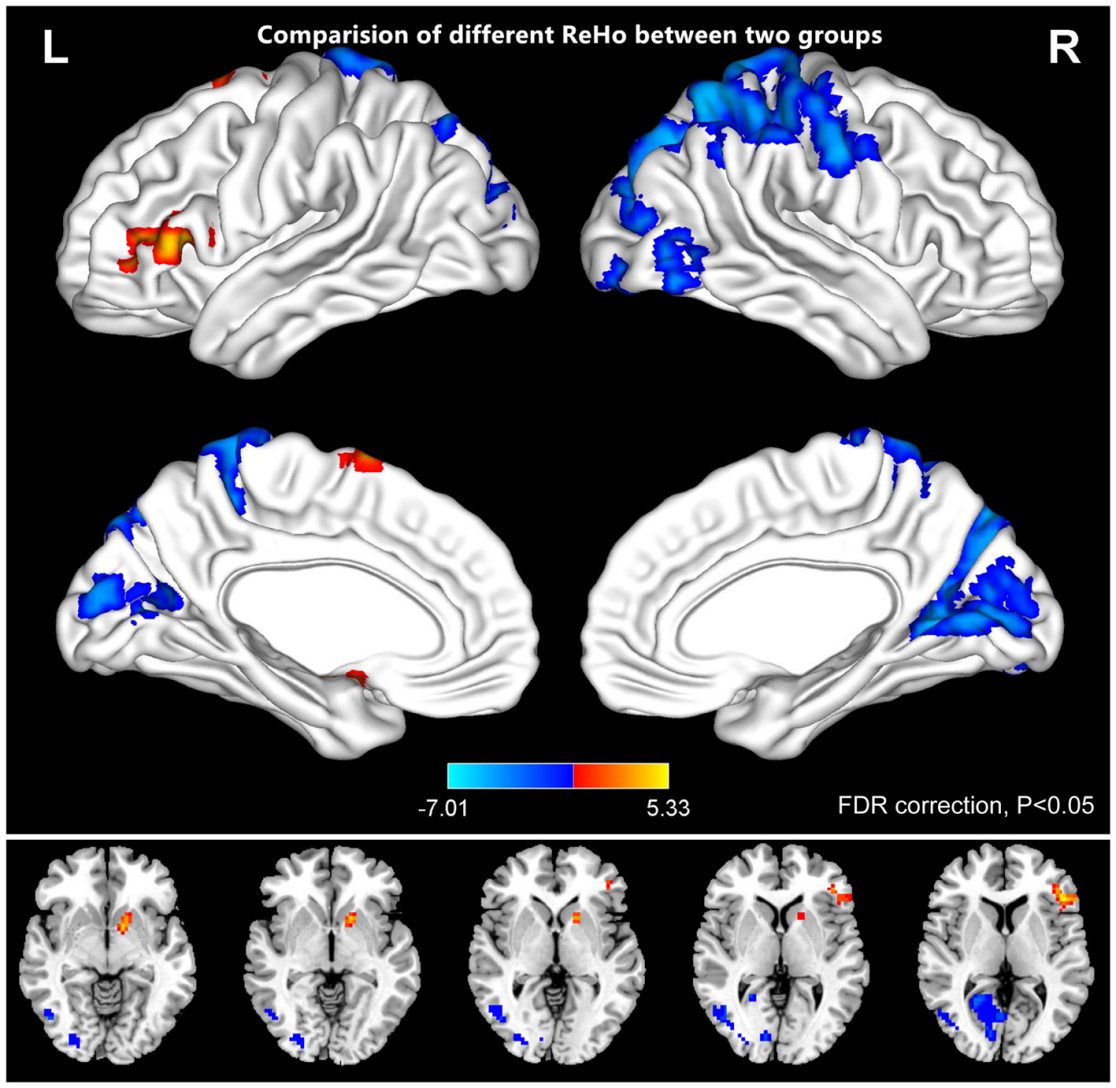
The comparison of different ReHo between the patients of iridocyclitis and healthy volunteers. The iridocyclitis patients displayed significantly reduced ReHo values in the right inferior occipital gyrus, the right middle temporal gyrus, the bilateral calcarine, the right postcentral gyrus, the left superior occipital gyrus, and the left precuneus and displayed enhanced ReHo values in the right cerebellum, the left putamen, the left supplementary motor area, and the left inferior frontal gyrus compared with healthy volunteers (FDR correction, *P* < 0.05). Abbreviations: L, left; R, right; FDR, false discovery rate correction.

**Figure 4:**
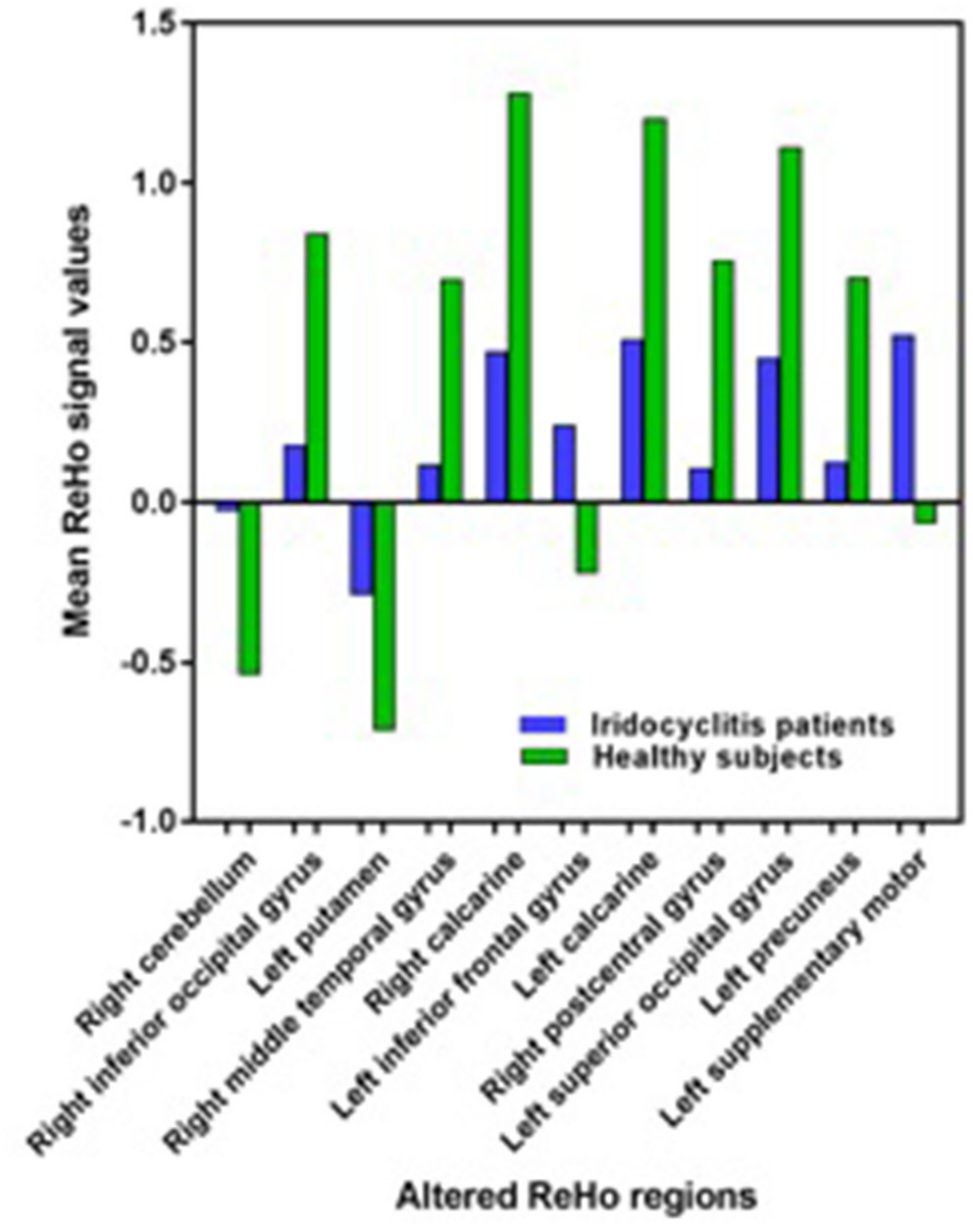
The average values of changed ReHo signal values between the iridocyclitis patients and healthy volunteers.

Figure 2 shows the spatial distributions of ReHo values of the patients with iridocyclitis and healthy volunteers. Compared with healthy volunteers, significantly decreased ReHo value was observed in the patients of iridocyclitis in the following brain regions: the right inferior occipital gyrus, the right middle temporal gyrus, the bilateral calcarine, the right postcentral gyrus, the left superior occipital gyrus, and the left precuneus [FDR correction, *P* < 0.05; Figure 3 (blue)]. In contrast, it was observed that the ReHo value was significantly enhanced in the right cerebellum, the left putamen, the left supplementary motor area, and the left inferior frontal gyrus in patients with iridocyclitis compared with healthy volunteers [FDR correction, *P* < 0.05; Figure 3 (red)]. Table 2 exhibited the altered brain areas and corresponding information between iridocyclitis group and healthy volunteer group (FDR correction, *P* < 0.05). The average values of alterations in ReHo between the two groups are displayed as a histogram (Figure 4). Nevertheless, no notable correlation was observed between average ReHo values in altered brain areas and patients' clinical features (*P* > 0.05).

**Table 2:** Brain regions with significantly different ReHo signal values between the iridocyclitis patients and healthy volunteers.

**Conditions**	**Brain regions**	**Cluster size**	**MNI coordinates**			***t*-score of peak voxel**
			***X***	***Y***	***Z***	
IC < HCs	Right inferior occipital gyrus	37	27	−87	−3	−3.8763
IC < HCs	Right middle temporal gyrus	64	45	−69	−6	−4.0949
IC < HCs	Right calcarine	268	24	−57	9	−5.2475
IC < HCs	Left calcarine	30	−12	−63	18	−4.2266
IC < HCs	Right postcentral gyrus	1,234	30	−66	42	−7.0129
IC < HCs	Left superior occipital gyrus	88	−21	−87	18	−4.5975
IC < HCs	Left precuneus	118	−12	−42	57	−4.5016
IC > HCs	Right cerebellum	157	18	−42	−39	5.1730
IC > HCs	Left putamen	54	−12	3	−9	4.9854
IC > HCs	Left inferior frontal gyrus	96	−45	27	9	5.3275
IC > HCs	Left supplementary motor area	58	−9	6	63	4.2742

*x, y, and z are the locations of the peak voxels in standard MNI coordinates. The statistical threshold was set at P < 0.05 after FDR correction. Abbreviations: IC, iridocyclitis; MNI, Montreal Neurological Institute*.

### ROC Curve

**Figure 5:**
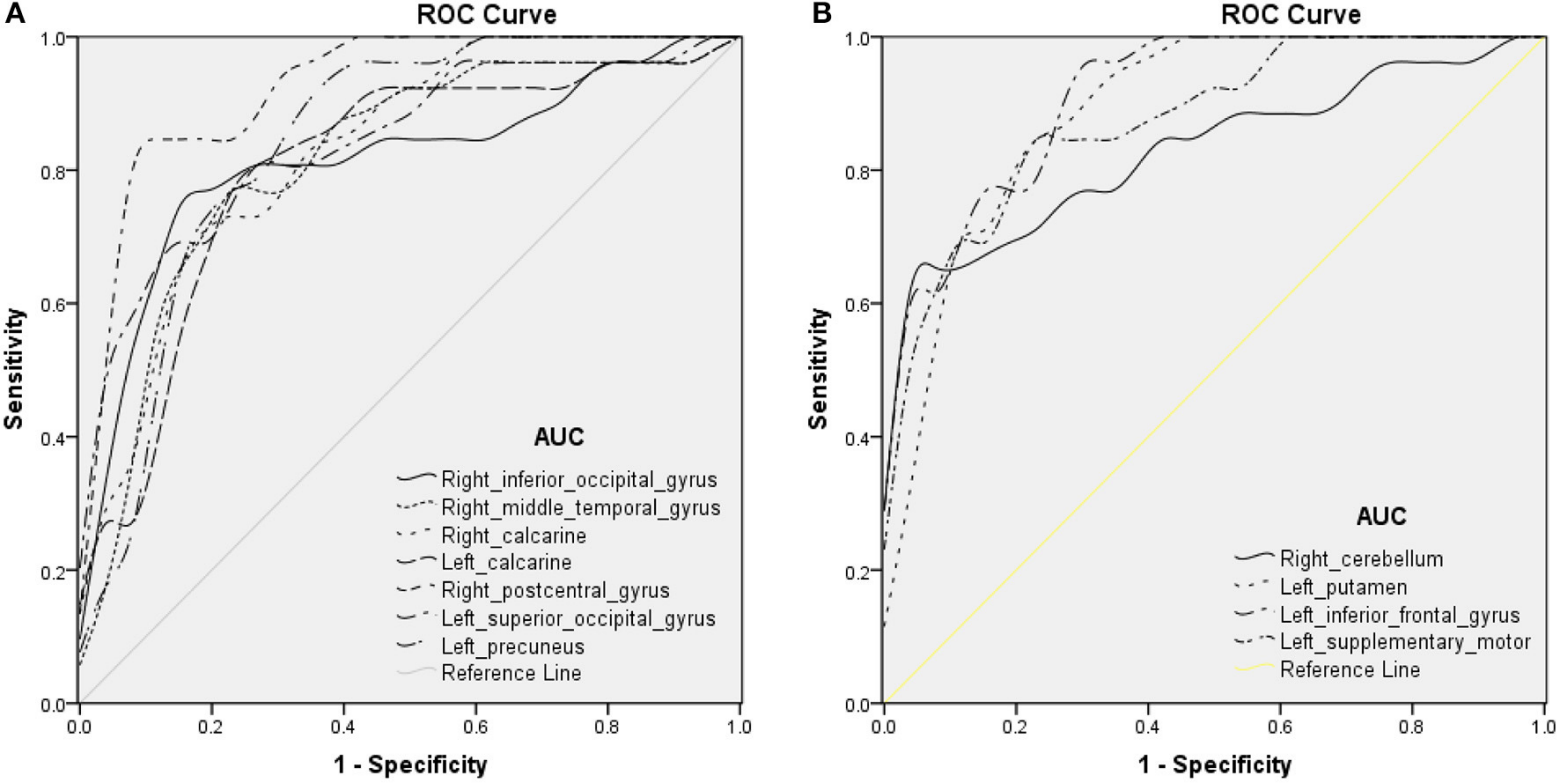
ROC curve analysis of the average ReHo of altered brain areas. ROC curve in ReHo values: **(A)** iridocyclitis < healthy volunteers: right inferior occipital gyrus [0.815; *P* < 0.001; 95% confidence interval (CI), 0.693–0.937], right middle temporal gyrus (0.815; *P* < 0.001; 95% CI, 0.695–0.936), right calcarine (0.834; *P* < 0.001; 95% CI, 0.726–0.942), left calcarine (0.806; *P* < 0.001; 95% CI, 0.683–0.930), right postcentral gyrus (0.926; *P* < 0.001; 95% CI, 0.854–0.998), left superior occipital gyrus (0.845; *P* < 0.001; 95% CI, 0.738–0.952), and left precuneus (0.843; *P* < 0.001; 95% CI, 0.733–0.954); **(B)** iridocyclitis > healthy volunteers: right cerebellum (0.833; *P* < 0.001; 95% CI, 0.719–0.947), left putamen (0.891; *P* < 0.001; 95% CI, 0.802–0.979), left inferior frontal gyrus (0.916; *P* < 0.001; 95% CI, 0.844–0.987), and left supplementary motor area (0.879; *P* < 0.001; 95% CI, 0.788–0.969). Abbreviations: IC, iridocyclitis; AUC, area under the ROC curve.

To explore whether the distinctive ReHo signal values obtained from the two groups could be a useful diagnostic marker to distinguish patients with iridocyclitis from healthy volunteers, ROC curve analysis was conducted. The areas under the ROC curve (AUCs) were as follows: right inferior occipital gyrus [0.815; *P* < 0.001; 95% confidence interval (CI), 0.693–0.937]; right middle temporal gyrus (0.815; *P* < 0.001; 95% CI, 0.695–0.936), right calcarine (0.834; *P* < 0.001; 95% CI, 0.726–0.942), left calcarine (0.806; *P* < 0.001; 95% CI, 0.683–0.930), right postcentral gyrus (0.926; *P* < 0.001; 95% CI, 0.854–0.998), left superior occipital gyrus (0.845; *P* < 0.001; 95% CI, 0.738–0.952), and left precuneus (0.843; *P* < 0.001; 95% CI, 0.733–0.954) (Table 3 and Figure 5A, iridocyclitis < healthy volunteers); right cerebellum (0.833; *P* < 0.001; 95% CI, 0.719–0.947); left putamen (0.891; *P* < 0.001; 95% CI, 0.802–0.979); left inferior frontal gyrus (0.916; *P* < 0.001; 95% CI, 0.844–0.987); and left supplementary motor area (0.879; *P* < 0.001; 95% CI, 0.788–0.969) (Table 3 and Figure 5B).

**Table 3:** ROC curves for the mean ReHo of changed brain areas.

**Conditions**	**Brain regions**	**AUC**	***P* values**	**95% CI**
IC < Healthy volunteers	Right inferior occipital gyrus	0.815	<0.001	0.693–0.937
IC < Healthy volunteers	Right middle temporal gyrus	0.815	<0.001	0.695–0.936
IC < Healthy volunteers	Right calcarine	0.834	<0.001	0.726–0.942
IC < Healthy volunteers	Left calcarine	0.806	<0.001	0.683–0.930
IC < Healthy volunteers	Right postcentral gyrus	0.926	<0.001	0.854–0.998
IC < Healthy volunteers	Left superior occipital gyrus	0.845	<0.001	0.738–0.952
IC < Healthy volunteers	Left precuneus	0.843	<0.001	0.733–0.954
IC > Healthy volunteers	Right cerebellum	0.833	<0.001	0.719–0.947
IC > Healthy volunteers	Left putamen	0.891	<0.001	0.802–0.979
IC > Healthy volunteers	Left inferior frontal gyrus	0.916	<0.001	0.844–0.987
IC > Healthy volunteers	Left supplementary motor area	0.879	<0.001	0.788–0.969

*Abbreviations: IC, iridocyclitis; AUC, area under the ROC curve; CI, confidence interval*.

## Discussion

**Figure 6:**
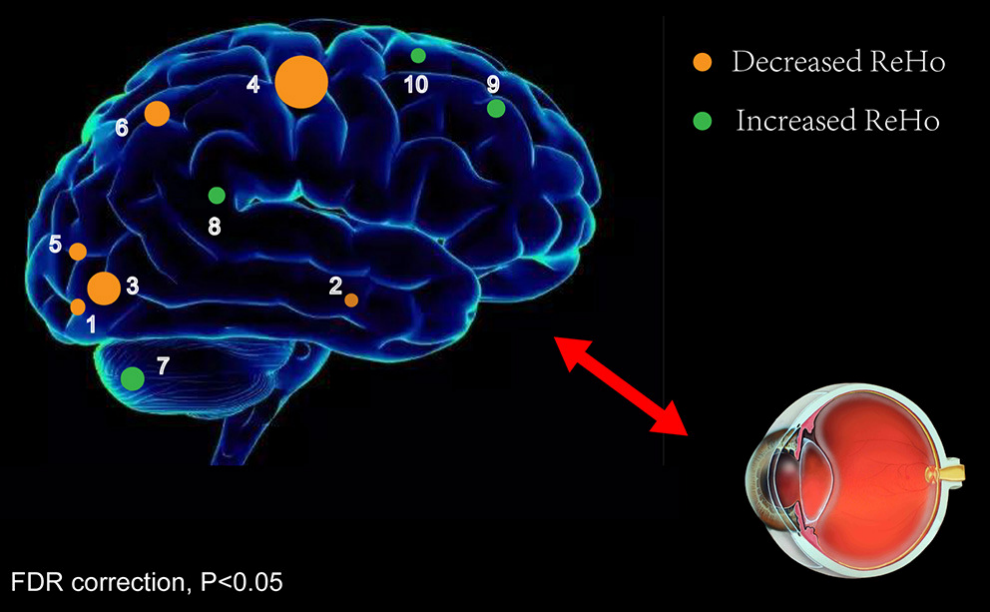
ReHo results of spontaneous neuronal activity in the iridocyclitis group. Compared to the healthy volunteers, the ReHo value of iridocyclitis patients in regions 1–6 was decreased to various extents, while the value of regions 7–10 was increased: [1] right inferior occipital gyrus (*t* = −3.8763), [2] right middle temporal gyrus (*t* = −4.0949), [3] right calcarine (*t* = −5.2475), [3] left calcarine (*t* = −4.2266), [4] right postcentral gyrus (*t* = −7.0129), [5] left superior occipital gyrus (*t* = −4.5975), [6] left precuneus (*t* = −4.5016), [7] right cerebellum (*t* = 5.1730), [8] left putamen (*t* = 4.9854), [9] left inferior frontal gyrus (*t* = 5.3275), and [10] left supplementary motor area (*t* = 4.2742). Note: Spot sizes indicate degrees of quantitative changes. Abbreviations: ReHo, regional homogeneity; FDR, false discovery rate correction.

ReHo values represent the local spontaneous coherence of neural activity and have been widely applied for analysis of multiple ophthalmologic diseases (23, 26, 33), such that this technique has considerable potential (Table 4). To our knowledge, it is the first study in which the ReHo technique has been applied to evaluate the effect of iridocyclitis on resting-state synchronous brain activity. The results of this study exhibited that, compared with healthy volunteers, patients with iridocyclitis had reduced ReHo in the right inferior occipital gyrus, bilateral calcarine, right middle temporal gyrus, left superior occipital gyrus, right postcentral gyrus, and left precuneus. They also had enhanced ReHo in the right cerebellum, left putamen, left inferior frontal gyrus, and left supplementary motor area (Figure 6).

**Table 4:** Regional homogeneity method applied in ophthalmological diseases.

**First author**	**Year**	**Disease**	**References**
Chen et al.	2017	Glaucoma	(34)
Dan et al.	2019	Retinitis pigmentosa	(29)
Shao et al.	2015	Optic neuritis	(35)
Huang et al.	2017	Retinal detachment	(26)
Huang et al.	2016	Concomitant strabismus	(24)
Huang et al.	2017	Late monocular blindness	(36)
Yang et al.	2019	Amblyopia	(37)
Liao et al.	2018	Diabetic retinopathy	(23)
Tang et al.	2018	Eye pain	(33)

We found that patients with iridocyclitis generally exhibited significant reduction of ReHo values in portions of vision-related regions. The occipital lobe is a crucial anatomical area for visual information processing. It also controls pupil accommodation reflex activities and eye movements related to vision (38). The calcarine sulcus is located in the medial surface of the occipital lobe, within the primary visual cortex (V1). Retinal photoreceptors take a core role in visual function. They convert light signals to nerve impulses and transmit these impulses to retinal ganglion cells. Numerous studies have shown that through the visual pathway, visual signals are projected to the visual cortex (39). Although iridocyclitis is defined as inflammation of the iris and ciliary body, recent studies have demonstrated retinal involvement (8, 40, 41). These findings imply that simultaneous fluid accumulation in the retina and choroid during acute inflammation, combined with deprivation of retinal input, might lead to functional alterations within the visual cortex. Thus, we presume that visual information processing in the brain might have deteriorated in patients with iridocyclitis because of this vision loss.

The main clusters with decreased ReHo values were observed in the right postcentral gyrus. Anatomically, the postcentral gyrus belongs to the primary somatosensory cortex (S1), which receives somatosensory input from the thalamocortical systems and sends these inputs to other parts of the somatosensory cortex (42). The postcentral gyrus participates in various sensory perceptions (such as the temperature and position perceptions) and is also involved in the central processing of the pain, tactile stimuli, and sense of touch (43, 44). Some neuroimaging researches have reported that multiple pain-related diseases are related to S1 dysfunction, including acute eye pain and low back pain (33, 45). Consistent with those findings, the reduced ReHo values observed in our study indicate that patients with iridocyclitis may exhibit abnormal local synchronization in the postcentral gyrus due to clinical symptoms of chronic recurrent eye pain. Furthermore, the postcentral gyrus is reportedly strongly associated with spontaneous activity in the primary visual areas and is jointly activated with the occipital visual areas during visual imagery tasks (46). In support of these findings, we found patients with iridocyclitis showed lower ReHo area in the right postcentral gyrus compared to healthy volunteers, which may suggest a harmful effect on the postcentral gyrus.

The functions of the middle temporal gyrus are complex and diverse. It participates in the composition of the visual ventral processing stream and primary auditory projection, as well as in brain functional activities such as visual memory and semantic processing (47). The results of the present study showed that iridocyclitis may influence the visual memory functions of affected patients. In addition, the middle temporal gyrus is a critical component of the default mode network, which is primarily activated in the resting state and exhibits reduced activity in the task-based state. The default mode network is related to cognition, emotional processing, self-reflection, and memory; its dysfunction has been observed in many diseases, such as depression and Alzheimer's disease (48, 49). Thus far, several studies have explored vision-related quality of life and mental health status in patients with uveitis (13, 14). Qian et al. found that depression is a major comorbidity in patients with ocular inflammatory disease, while Hoeksema et al. reported that patients with HLA-B27-anterior uveitis exhibited more depressive symptoms and negative coping strategies, compared with controls (15, 50). In this study, the results displayed reduced ReHo values in the middle temporal gyrus in patients with iridocyclitis, compared with healthy volunteers, suggesting that patients with iridocyclitis might exhibit dysfunction in terms of cognition and emotion regulation.

As a component of the superior parietal lobule and the core of the frontoparietal central-executive network, the precuneus connects with the adjacent visual cortical regions and with visual areas in the cuneus (51). Utevsky et al. demonstrated that the precuneus also serves as a critical component of the default mode network (52). Functionally, the precuneus has a key part in various highly integrated tasks, such as visuomotor coordination, episodic memory retrieval, visuospatial imagery, as well as working memory (53, 54). Thus, the reduction of ReHo signal values in the precuneus might reflect impaired precuneus function in the iridocyclitis group.

Notably, we observed that patients with iridocyclitis displayed enhanced ReHo values in regions of the right cerebellum. This brain area participates in multiple functions, especially motor control. Damage to the cerebellum may result in dysfunctions of movement, affective regulation, and visuomotor coordination (55, 56). Iridocyclitis is known to be the foremost clinical characteristic of ankylosing spondylitis in a subset of patients. Li et al. found that patients with ankylosing spondylitis exhibited enhanced activation in the cerebellum anterior lobe on fMRI (57). Consistent with those findings, we also observed patients with iridocyclitis exhibited increased ReHo values in the cerebellum. Therefore, we hypothesize iridocyclitis may contribute to compensatory motor function enhancement in the cerebellum.

The putamen is a large nucleus of the basal ganglia that participates in motor control and constitutes a core component of the basal ganglia network (58). Furthermore, the putamen is closely associated with learning (59). We observed that patients with iridocyclitis exhibited enhanced ReHo values in the left putamen. Therefore, we speculate iridocyclitis might contribute to functional alteration of the putamen. The finding of enhanced spontaneous neuronal activity in the left supplementary motor area further indicates the potential compensatory mechanism of the motor function in the patients of iridocyclitis.

The ROC curve indicates the reliability of the results. AUC values of 0.7–0.9 are presumed to indicate perfect accuracy, values of 0.5–0.7 are considered moderate accuracy, and values <0.5 are considered low accuracy. The ROC curve analysis in our study revealed that AUCs in each brain area exceeded 0.8, which suggested that those specific ReHo differences had high diagnostic accuracy in identification of iridocyclitis. In summary, our results indicate that the ReHo method might constitute a sensitive fMRI measurement for the future diagnosis of patients with iridocyclitis.

There were several limitations in this study. First, the impacts of physiological noise (e.g., respiratory fluctuations, head motion, and cardiac fluctuation) were not completely eliminated and might reduce the specificity of the results. To improve the reliability of ReHo, careful optimization and preprocessing of the data (such as linear regression analysis) can be performed. Second, relatively minimal data were included in the analysis, which may have restricted the generalizability of the results and the corresponding statistical power. In a future study, we will include additional data and conduct a multicenter investigation to verify the current findings. Further parameters, including comprehensive clinical assessments and the duration of iridocyclitis in affected patients, will also be included in the correlation analysis. Third, in addition to spontaneous neural activity in the brain measured by ReHo, multimodal MRI imaging technologies should be applied to further investigate the brain function alterations in individuals with iridocyclitis.

## Conclusion

Our study demonstrated that patients with iridocyclitis exhibited disturbed synchronous neural activities in specific brain areas, including the visual, motor, and somatosensory regions, as well as the default mode network, compared with healthy volunteers. These results might offer valuable information for use in investigation of the neuropathological or compensatory mechanisms in patients with iridocyclitis and suggest a potential approach for further treatment development.

## Data Availability Statement

The original contributions presented in the study are included in the article/supplementary material, further inquiries can be directed to the corresponding author/s.

## Ethics Statement

The studies involving human participants were reviewed and approved by Renmin Hospital of Wuhan University. The patients/participants provided their written informed consent to participate in this study.

## Author Contributions

YT contributed to study design, fMRI data analysis, and drafting the manuscript. XH contributed to design the protocol and data collection. C-XQ contributed to data collection and manuscript discussion. YS conceived the study, reviewed, and revised the manuscript. All authors read and approved the final manuscript.

## Conflict of Interest

The authors declare that the research was conducted in the absence of any commercial or financial relationships that could be construed as a potential conflict of interest.

## References

[1] Al-Ani HH Sims JL Tomkins-Netzer O Lightman S Niederer RL. Vision loss in anterior uveitis. Brit J Ophthalmol. (2020) 12:104. 10.1136/bjophthalmol-2019-315551 32245851

[2] Niccoli L Nannini C Cassarà E Kaloudi O Susini M Lenzetti I. Frequency of iridocyclitis in patients with early psoriatic arthritis: a prospective, follow up study. Int J Rheum Dis. (2012) 15:414–8. 10.1111/j.1756-185X.2012.01736.x 22898222

[3] Chang JH McCluskey PJ Wakefield D. Acute anterior uveitis and HLA-B27. Surv Ophthalmol. (2005) 50:364–88. 10.1016/j.survophthal.2005.04.003 15967191

[4] Menezo V Lightman S. The development of complications in patients with chronic anterior uveitis. Am J Ophthalmol. (2005) 139:988–92. 10.1016/j.ajo.2005.01.029 15953427

[5] Forrester JV Kuffova L Dick AD. Autoimmunity, Autoinflammation, and infection in uveitis. Am J Ophthalmol. (2018) 189:77–85. 10.1016/j.ajo.2018.02.019 29505775

[6] Asrani S Moore DB Jaffe GJ. Paradoxical changes of retinal nerve fiber layer thickness in uveitic glaucoma. JAMA Ophthalmol. (2014) 132:877–80. 10.1001/jamaophthalmol.2014.954 24852038

[7] Din NM Taylor SRJ Isa H Tomkins-Netzer O Bar A Talat L. Evaluation of retinal nerve fiber layer thickness in eyes with hypertensive uveitis. JAMA Ophthalmol. (2014) 132:859–65. 10.1001/jamaophthalmol.2014.404 24789528

[8] Traill A Stawell R Hall A Zamir E. Macular thickening in acute anterior uveitis. Ophthalmology. (2007) 114:2. 10.1016/j.ophtha.2006.07.028 17270703

[9] Tripathi P Saxena S Yadav VS Naik S Singh VK. Human S-antigen: peptide determinant recognition in uveitis patients. Exp Mol Pathol. (2004) 76:122–8. 10.1016/j.yexmp.2003.10.007 15010290

[10] Kim M Choi SY Park Y-H. Analysis of choroidal and central foveal thicknesses in acute anterior uveitis by enhanced-depth imaging optical coherence tomography. BMC Ophthalmol. (2017) 17:225. 10.1186/s12886-017-0628-7 29191218 PMC5709927

[11] Pichi F Sarraf D Arepalli S Lowder CY Cunningham ET Neri P. The application of optical coherence tomography angiography in uveitis and inflammatory eye diseases. Prog Retin Eye Res. (2017) 59:178–201. 10.1016/j.preteyeres.2017.04.005 28465249

[12] Cerquaglia A Iaccheri B Fiore T Fruttini D Belli FB Khairallah M. New insights on ocular sarcoidosis: an optical coherence tomography angiography study. Ocul Immunol Inflamm. (2018) 27:1057–66. 10.1080/09273948.2018.1497665 30081683

[13] Franke GH Schütte E Heiligenhaus A. Psychosomatik der uveitis - eine pilotstudie. Psychother Psychosom Med Psychol. (2005) 55:65–71. 10.1055/s-2004-828504 15702425

[14] Scott IU. Visual functioning and general health status in patients with uveitis. Evidence-Based Eye Care. (2002) 3:92–3. 10.1097/00132578-200204000-0001511405835

[15] Maca SM Schiesser AW Sobala A Gruber K Pakesch G Prause C. Distress, depression and coping in HLA-B27-associated anterior uveitis with focus on gender differences. Brit J Ophthalmol. (2010) 95:699–704. 10.1136/bjo.2009.174839 20971789

[16] Biswal BB. Resting state fMRI: a personal history. NeuroImage. (2012) 62:938–44. 10.1016/j.neuroimage.2012.01.090 22326802 PMC12911935

[17] Sturm AK König P. Mechanisms to synchronize neuronal activity. Biol Cybern. (2001) 84:153–72. 10.1007/s004220000209 11252634

[18] Jutras MJ Buffalo EA. Synchronous neural activity and memory formation. Curr Opin Neurobiol. (2010) 20:150–5. 10.1016/j.conb.2010.02.006 20303255 PMC2862842

[19] Bayati M Valizadeh A Abbassian A Cheng S. Self-organization of synchronous activity propagation in neuronal networks driven by local excitation. Front Comput Neurosci. (2015) 9:69. 10.3389/fncom.2015.00069 26089794 PMC4454885

[20] Zang Y Jiang T Lu Y He Y Tian L. Regional homogeneity approach to fMRI data analysis. NeuroImage. (2004) 22:394–400. 10.1016/j.neuroimage.2003.12.030 15110032

[21] Tononi G McIntosh AR Russell DP Edelman GM. Functional clustering: identifying strongly interactive brain regions in neuroimaging data. NeuroImage. (1998) 7:133–49. 10.1006/nimg.1997.0313 9558645

[22] Li Z Kadivar A Pluta J Dunlop J Wang Z. Test-retest stability analysis of resting brain activity revealed by blood oxygen level-dependent functional MRI. J Magn Reson Imaging. (2012) 36:344–54. 10.1002/jmri.23670 22535702 PMC3399952

[23] Liao X-L Yuan Q Shi W-Q Li B Su T Lin Q. Altered brain activity in patients with diabetic retinopathy using regional homogeneity: a resting-state fMRI study. Endocr Pract. (2019) 25:320–7. 10.4158/ep-2018-0517 30995427

[24] Shao Y Huang X Li S-H Zhou F-Q Zhang Y Zhong Y-L. Altered intrinsic regional brain spontaneous activity in patients with comitant strabismus: a resting-state functional MRI study. Neuropsychiatr Dis Treat. (2016) 12:1303–8. 10.2147/ndt.S105478 27350747 PMC4902244

[25] Chen K Lin X Ding K Liu Y Yan X Song S. Altered spontaneous activity in anisometropic amblyopia subjects: revealed by resting-state fMRI. PLoS ONE. (2012) 7:e43373. 10.1371/journal.pone.0043373 22937041 PMC3427333

[26] Huang X Li D Li H-J Zhong Y-L Freeberg S Bao J. Abnormal regional spontaneous neural activity in visual pathway in retinal detachment patients: a resting-state functional MRI study. Neuropsychiatr Dis Treat. (2017) 13:2849–54. 10.2147/ndt.S147645 29200859 PMC5703148

[27] Standardization of Uveitis Nomenclature for Reporting Clinical Data. Results of the First International Workshop. Am J Ophthalmol. (2005) 140:509–16. 10.1016/j.ajo.2005.03.057 16196117 PMC8935739

[28] Ni M-F Zhang B-W Chang Y Huang X-F Wang X-M. Altered resting-state network connectivity in panic disorder: an independent component analysis. Brain Imaging Behav. (2020) 14. 10.1007/s11682-020-00329-z 32748315

[29] Dan H-D Zhou F-Q Huang X Xing Y-Q Shen Y. Altered intra- and inter-regional functional connectivity of the visual cortex in individuals with peripheral vision loss due to retinitis pigmentosa. Vision Res. (2019) 159:68–75. 10.1016/j.visres.2019.02.013 30904614

[30] Yan C-G Wang X-D Zuo X-N Zang Y-F. DPABI: data processing & analysis for (resting-state) brain imaging. Neuroinformatics. (2016) 14:339–51. 10.1007/s12021-016-9299-4 27075850

[31] Goto M Abe O Aoki S Hayashi N Miyati T Takao H. Diffeomorphic anatomical registration through exponentiated lie algebra provides reduced effect of scanner for cortex volumetry with atlas-based method in healthy subjects. Neuroradoilogy. (2013) 55:869–75. 10.1007/s00234-013-1193-2 23619702

[32] Lowe MJ Mock BJ Sorenson JA. Functional Connectivity in single and multislice echoplanar imaging using resting-state fluctuations. NeuroImage. (1998) 7:119–32. 10.1006/nimg.1997.0315 9558644

[33] Tang L-Y Li H-J Huang X Bao J Sethi Z Ye L. Assessment of synchronous neural activities revealed by regional homogeneity in individuals with acute eye pain: a resting-state functional magnetic resonance imaging study. J Pain Res. (2018) 11:843–50. 10.2147/jpr.S156634 29719418 PMC5916265

[34] Chen W Zhang L Xu Y-g Zhu K Luo M. Primary angle-closure glaucomas disturb regional spontaneous brain activity in the visual pathway: an fMRI study. Neuropsychiatr Dis Treat. (2017) 13:1409–17. 10.2147/ndt.S134258 28579788 PMC5449125

[35] Shao Y Cai F Zhong Y Huang X Zhang Y Hu P-H. Altered intrinsic regional spontaneous brain activity in patients with optic neuritis: a resting-state functional magnetic resonance imaging study. Neuropsychiatr Dis Treat. (2015) 11:3065–73. 10.2147/ndt.S92968 26715848 PMC4686319

[36] Huang X Ye C-L Zhong Y-L Ye L Yang Q-C Li H-J. Altered regional homogeneity in patients with late monocular blindness. Neuroreport. (2017) 28:1085–91. 10.1097/wnr.0000000000000855 28858036 PMC5916480

[37] Yang X Lu L Li Q Huang X Gong Q Liu L. Altered spontaneous brain activity in patients with strabismic amblyopia: a resting-state fMRI study using regional homogeneity analysis. Exp Ther Med. (2019) 18:3877–84. 10.3892/etm.2019.8038 31616514 PMC6781806

[38] Shao Y Li QH Li B Lin Q Su T Shi WQ. Altered brain activity in patients with strabismus and amblyopia detected by analysis of regional homogeneity: a resting-state functional magnetic resonance imaging study. Mol Med Report. (2019) 19:4832–40. 10.3892/mmr.2019.10147 PMC6522834 31059016

[39] Hadjikhani N Tootell RBH. Projection of rods and cones within human visual cortex. Hum Brain Mapp. (2000) 9:55−63. 10.1002/(sici)1097-0193(2000)9:1<55::Aid-hbm6>3.0.co;2-u 10643730 PMC6871842

[40] Castellano CG Stinnett SS Mettu PS McCallum RM Jaffe GJ. Retinal thickening in iridocyclitis. Am J Ophthalmol. (2009) 148:341–9.e1. 10.1016/j.ajo.2009.03.034 19477710

[41] Géhl Z Kulcsár K Kiss HJM Németh J Maneschg OA Resch MD. Retinal and choroidal thickness measurements using spectral domain optical coherence tomography in anterior and intermediate uveitis. BMC Ophthalmol. (2014) 14:103. 10.1186/1471-2415-14-103 25176513 PMC4236668

[42] Wang Z Chen Li M Négyessy L Friedman Robert M Mishra A Gore John C. The relationship of anatomical and functional connectivity to resting-state connectivity in primate somatosensory cortex. Neuron. (2013) 78:1116–26. 10.1016/j.neuron.2013.04.023 23791200 PMC3723346

[43] Mori K Osada H Yamamoto T Nakao Y Maeda M. Pterional keyhole approach to middle cerebral artery aneurysms through an outer canthal skin incision. Minim Invasive Neurosurg. (2007) 50:195–201. 10.1055/s-2007-985837 17948177

[44] Wrigley PJ Press SR Gustin SM Macefield VG Gandevia SC Cousins MJ . Neuropathic pain and primary somatosensory cortex reorganization following spinal cord injury. Pain. (2009) 141:52–9. 10.1016/j.pain.2008.10.007 19027233

[45] Frot M Magnin M Mauguière F Garcia-Larrea L. Cortical representation of pain in primary sensory-motor areas (S1/M1)-a study using intracortical recordings in humans. Hum Brain Mapp. (2013) 34:2655–68. 10.1002/hbm.22097 22706963 PMC6869910

[46] Wang K Jiang T Yu C Tian L Li J Liu Y. Spontaneous activity associated with primary visual cortex: a resting-state fMRI study. Cereb Cortex. (2007) 18:697–704. 10.1093/cercor/bhm105 17602140

[47] Visser M Jefferies E Embleton KV Lambon Ralph MA. Both the middle temporal gyrus and the ventral anterior temporal area are crucial for multimodal semantic processing: distortion-corrected fMRI evidence for a double gradient of information convergence in the temporal lobes. J Cog Neurosci. (2012) 24:1766–78. 10.1162/jocn_a_00244 22621260

[48] Yu-Feng Z Yong H Chao-Zhe Z Qing-Jiu C Man-Qiu S Meng L. Altered baseline brain activity in children with ADHD revealed by resting-state functional MRI. Brain Dev. (2007) 29:83–91. 10.1016/j.braindev.2006.07.002 16919409

[49] Weissman-Fogel I Moayedi M Taylor KS Pope G Davis KD. Cognitive and default-mode resting state networks: do male and female brains “rest” differently? Hum Brain Mapp. (2010) 11:1713–26. 10.1002/hbm.20968 20725910 PMC6870948

[50] Qian Y Glaser T Esterberg E Acharya NR. Depression and visual functioning in patients with ocular inflammatory disease. Am J Ophthalmol. (2012) 153:370–8.e2. 10.1016/j.ajo.2011.06.028 21924399 PMC3243823

[51] Cavanna AE Trimble MR. The precuneus: a review of its functional anatomy and behavioural correlates. Brain. (2006) 129:564–83. 10.1093/brain/awl004 16399806

[52] Utevsky AV Smith DV Huettel SA. Precuneus is a functional core of the default-mode network. J Neurosci. (2014) 34:932–0. 10.1523/jneurosci.4227-13.2014 24431451 PMC3891968

[53] Wallentin M Weed E Østergaard L Mouridsen K Roepstorff A. Accessing the mental space—spatial working memory processes for language and vision overlap in precuneus. Hum Brain Mapp. (2008) 29:524–32. 10.1002/hbm.20413 17525981 PMC6871041

[54] Wenderoth N Debaere F Sunaert S Swinnen SP. The role of anterior cingulate cortex and precuneus in the coordination of motor behaviour. Eur J Neuroscie. (2005) 22:235–46. 10.1111/j.1460-9568.2005.04176.x 16029213

[55] Noroozian M. The role of the cerebellum in cognition. Neurol Clin. (2014) 32:1081–104. 10.1016/j.ncl.2014.07.005 25439295

[56] Kralj-Hans I Baizer JS Swales C Glickstein M. Independent roles for the dorsal paraflocculus and vermal lobule VII of the cerebellum in visuomotor coordination. Exp Brain Res. (2006) 177:209–22. 10.1007/s00221-006-0661-x 16951960

[57] Li C Wei X Zou Q Zhang Y Yin X Zhao J. Cerebral functional deficits in patients with ankylosing spondylitis- an fMRI study. Brain Imaging Behav. (2016) 11:936–42. 10.1007/s11682-016-9565-y 27394669

[58] Vicente AF Bermudez MA Romero MdC Perez R Gonzalez F. Putamen neurons process both sensory and motor information during a complex task. Brain Res. (2012) 1466:70–81. 10.1016/j.brainres.2012.05.037 22640776

[59] Grahn JA Parkinson JA Owen AM. The role of the basal ganglia in learning and memory: neuropsychological studies. Behav Brain Res. (2009) 199:53–60. 10.1016/j.bbr.2008.11.020 19059285

